# Healthcare providers insights on the Baby-Friendly Hospital Initiative: A cross-sectional study in Qatar

**DOI:** 10.18332/ejm/203687

**Published:** 2025-06-12

**Authors:** Jussara D. S. Brito, Kalpana Singh, Laura Falcon, Soad Elkhaligy, Tamara Alshdafat, Salwa Alrawaili, Lolwa Alansari

**Affiliations:** 1 Al Wakra Hospital, Hamad Medical Corporation, Al Wakra, Qatar; 2 Nursing Researcher Department, Hamad Medical Corporation, Doha, Qatar; 3 Women’s Wellness & Research Centre, Hamad Medical Corporation, Doha, Qatar; 4 Gardenia Medical Center, Doha, Qatar

**Keywords:** Baby-Friendly Hospital Initiative, KAP analysis, breastfeeding, healthcare

## Abstract

**INTRODUCTION:**

The Baby-Friendly Hospital Initiative (BFHI) by WHO and UNICEF promotes exclusive breastfeeding and enhances maternal and child health. Introduced in Qatar in 2016, its accreditation and implementation remain incomplete. This study evaluates the knowledge, attitudes, and practices (KAP) of clinical staff regarding BFHI at Al-Wakra Hospital.

**METHODS:**

A cross-sectional study was conducted among clinical staff in Obstetrics, Gynecology, and Pediatrics at Al-Wakra Hospital, using a convenience sampling method from April 2022 to January 2023. An online questionnaire based on the WHO/UNICEF Competency Verification Toolkit gathered data on sociodemographic, WHO BFHI course participation, and KAP levels. Statistical analysis was performed using t-tests and ANOVA in STATA 17.0, with significance at p<0.05.

**RESULTS:**

The study involved 141 participants, predominantly female (87.2%) with an average age of 38.4 years; most were nurses (85.1%). Knowledge scores were higher in males (8.0 ± 1.3) than females (7.1 ± 1.8, p=0.051), and specialists scored better than nurses (8.0 ± 1.4 vs 7.1 ± 1.8, p=0.033). A weak correlation was found between age and KAP scores.

**CONCLUSIONS:**

The study highlights the need for continued BFHI education, especially for head nurses and midwives, to enhance implementation and ensure consistent competency among healthcare professionals.

## INTRODUCTION

Enhancing the health and well-being of women and children stands as a paramount objective in global political and social agendas^1^. The imperative to improve maternal and childcare has been consistently advocated by various international health organizations, such as the American Academy of Pediatrics (AAP) and the United Nations International Children’s Emergency Fund/World Health Organization (UNICEF/WHO)^1,2^. Breastfeeding emerges as a primary strategy toward achieving the goals of ensuring a healthy mother, baby and society. The Baby-Friendly Hospital Initiative (BFHI), initiated by UNICEF and WHO in 1991, signifies a global commitment to safeguard, promote, and support exclusive breastfeeding for the first six months of life, followed by continued breastfeeding up to two years alongside complementary foods^1^.

Despite evidence-based studies highlighting the benefits of breastfeeding for both infants and mothers as well as for the environment and society^3,4^, global breastfeeding rates remain suboptimal^5,6^. Only 41% of infants worldwide under the age of six months are exclusively breastfed, and merely 45% continue breastfeeding until two years of age^7,8^. The BFHI advocates for all maternal and child healthcare facilities to adopt the Ten Steps for Successful Breastfeeding and adhere to the International Code of Marketing of Breast Milk globally, with the specific aim of increasing breastfeeding rates, especially exclusive breastfeeding until six months of age^1^.

In Qatar, the implementation of the BFHI began in 2016, coinciding with the drafting of a bill regarding the marketing of breastmilk substitutes^9^.

A study by Abd El-Ghany et al.^10^ underscores the importance of self-appraisal among staff during the BFHI implementation process, indicating the need for insight into staff’s knowledge, attitudes, and practices. Recognizing the process of establish BFHI programs, legislations, or accreditations in Qatar, it becomes crucial to gain a comprehensive understanding of the current BFHI-related knowledge among staff, their attitudes, and their adherence to BFHI practices. The Baby-Friendly Hospital Initiative (BFHI) is a global program designed to promote and support breastfeeding in maternity facilities. This study conducts a comprehensive knowledge, attitudes, and practices (KAP) analysis among healthcare professionals to assess their awareness, perceptions, and adherence to the BFHI guidelines. By understanding the KAP of healthcare professionals, this research aims to identify potential gaps and opportunities for improvement in implementing BFHI practices.

## METHODS

### Study design

This cross-sectional study was conducted to assess the knowledge, attitudes, and practices (KAP) of clinical staff regarding their participation in the 20-hour WHO/UNICEF/BFHI course. The study explores potential factors, including sociodemographic characteristics such as age, gender, level of education, position, working areas (e.g. LR, NICU), and participation in the WHO BFHI 20-hour program.

### Sample size and sampling method

All staff stationed in the Obstetrics & Gynecology and Pediatrics departments at Al-Wakra Hospital, Qatar, were invited to participate. The inclusion criteria comprised staff from Al-Wakra public hospital in Qatar, specifically in Obstetrics & Gynecology, and Pediatrics Departments, actively involved in providing direct care, education, or assistance for women and/or children of breastfeeding age. This included Registered Nurses (RN), Midwives (MD), Charge Nurses (CN), Head Nurses (HN), Clinical Nurse Specialists (CNS), physicians (specialists and consultants), Lactation consultants (LC), and Nursing educators. Full and partial contract staff with access to facility email were also eligible. The study’s limited scope, focusing exclusively on Al-Wakra hospital staff, facilitated the distribution of an online structured self-administered questionnaire through hospital intra-mail groups.

### Instrument

The questionnaire, consisting of 31 questions derived from the WHO/UNICEF/BFHI Competency Verification Toolkit, covered four parts: Demographics and General Information (7 questions), Knowledge (11 questions), Attitude (5 questions), and Practice (9 questions)^11^. A pilot test involving 50 staff ensured questionnaire clarity, cultural appropriateness, and practicality. Revisions were made based on participant feedback regarding ease of administration, understandability, completion time estimation, question ambiguities, and data completeness.

### Ethical considerations

The study was carried out in adherence to the guidelines and principles set out in the Declaration of Helsinki. The study was approved by the IRB of … (Approval number: MRC-01-21-939). Informed consent was obtained from subjects who participated in the research.

### Statistical analysis

Descriptive statistics were used to summarize sociodemographic characteristics and KAP scores. Qualitative variables are presented as absolute (n) and relative frequencies (%), while quantitative variables are summarized using mean ± standard deviation (SD). Comparative analyses were conducted to examine differences in KAP scores across demographic subgroups. The chi-squared test (or Fisher’s exact test where applicable) was used for categorical variables. For continuous variables, independent t-tests were performed for two-group comparisons, and one-way ANOVA was used for multiple-group comparisons. To explore relationships between knowledge, attitude, and practice scores, Pearson’s correlation coefficient (r) was used. A p<0.05 was considered statistically significant, and the findings are reported with 95% confidence intervals. All statistical analyses were conducted using STATA 17.0 statistical software.

## RESULTS

Table 1 describes the demographic and professional characteristics of 141 participants, predominantly female (87.2%) with a mean age of 38.4 years. Most hold roles as head nurses, charge nurses, midwives, or registered nurses (85.1%), while 14.9% are specialists. Participants are evenly split between two departments: LR/OBS/GYN/ED (48.2%) and Pediatric units (51.8%). The average professional experience is 15.6 years, with 95% having uninterrupted experience. Regarding breastfeeding training, 31.2% have completed the WHO 20-hour breastfeeding promotion and support training, and 97.2% reported that their facility has a breastfeeding policy in place.

**Table 1:** Sociodemographic characteristics of participants (N=141)

*Characteristics*	*Category*	*n (%)*
**Gender**	Female	123 (87.2)
	Male	18 (12.8)
**Age**, mean (SD)		38.4 (6.0)
**Position** (Your occupation/position)	Head/charge/midwife/RN	120 (85.1)
	Specialist/consultant/CNS	21 (14.9)
**Department** (You belong to which of the following departments)	LR/OBS/GYN/ED	68 (48.2)
	Pediatric-ED/IPW/PICU/NICU	73 (51.8)
**Professional experience** (years), mean (SD)		15.6 (6.7)
**Professional experience**	Interrupted	6 (4.3)
	Uninterrupted	134 (95.0)
**Facility has a breastfeeding policy**	No	4 (2.8)
	Yes	137 (97.2)
**Received the WHO 20-h breastfeeding promotion and support in a baby-friendly training**	No	95 (67.4)
	Yes	46 (32.6)

Responses to the questionnaire are detailed in Table 2. For knowledge questions, correct responses were highest for the reasons behind 24-hour rooming-in support for breastfeeding (95%), immediate skin-to-skin contact after birth (92.2%), and the time to interrupt skin-to-skin in the first two hours (85.1%). However, only 24.1% correctly identified global breastfeeding duration recommendations. In attitude questions, the highest correct responses where about how direct care providers can help avoid of promotion of bottles (83.7%) and the refusal of financial inducements (81%). The lowest correct responses were regarding responses to informational materials from infant feeding company representatives (13.5%). For practice questions, effective positioning of the baby at the breast and hand expression reminders had the highest correct responses (72.3%). Conversely, the lowest responses were about assessing newborns during skin-to-skin contact (13.5%) and handling thawed expressed breast milk (15.6%).

**Table 2:** Frequency of correct and false responses to the questions

*Domains and questions*	*Correct n (%)*	*False n (%)*
**Knowledge**		
Which item is covered by the International Code?	69 (48.9)	68 (48.2)
Which statement about exclusive breastfeeding is correct?	88 (62.4)	52 (36.9)
What is the global recommendation for duration of exclusive breastfeeding?	99 (70.2)	41 (29.1)
What is the global recommendation for how long a baby should be breastfed?	34 (24.1)	106 (75.2)
Why is breastfeeding important for the mother?	130 (92.2)	7 (5.0)
What is an important reason for immediate and sustained mother-baby skin-to-skin contact after birth?	130 (92.2)	7 (5.0)
Why should skin-to-skin be uninterrupted?	65 (46.1)	73 (51.8)
When would it be acceptable to interrupt skin-to-skin within the first 2 hours after birth?	120 (85.1)	17 (12.1)
When the baby is placed skin-to-skin on the mother at birth, what behaviors should he demonstrate instinctually before latching?	50 (35.5)	87 (61.7)
Why does 24-hour rooming-in support breastfeeding?	134 (95.0)	4 (2.8)
What does responsive/demand feed mean?	101 (71.6)	36 (25.5)
**Attitude**		
How should a direct care provider respond if offered informational materials provided by an infant feeding company representative?	19 (13.5)	117 (83.0)
How should a direct care provider respond if offered gifts provided by an infant feeding company representative?	112 (79.4)	23 (16.3)
Why should direct care providers refuse financial or material inducements?	114 (80.9)	20 (14.2)
How can direct care providers help or influence facilities to avoid deliberate or accidental promotion of feeding bottles or teats?	118 (83.7)	16 (11.3)
A breastfeeding mother is concerned about her 2-day old baby’s frequent crying. She asks to get a bottle of formula so that she and her baby can have a good sleep. What is your best action after you have listened to her concerns?	39 (27.7)	95 (67.4)
**Practice**		
What is the recommended sequence when placing the infant skin-to-skin with the mother?	87 (61.7)	45 (31.9)
What are key points that should be assessed when the newborn is skin-to-skin with the mother?	19 (13.5)	112 (79.4)
Which sequence best describes at least 3 aspects of safe care of the newborn in the first 2 hours after birth?	79 (56.0)	52 (36.9)
What are 2 things that should be observed when assessing a full breastfeeding session?	33 (23.4)	97 (68.8)
What is the best way to help a mother achieve a comfortable and safe position for breastfeeding during the hospital stay?	49 (34.8)	82 (58.2)
What are 2 key points for effective positioning baby at breast?	102 (72.3)	29 (20.6)
When demonstrating to a mother how to hand express her milk, it is important to remind her to:	104 (73.8)	25 (17.7)
Which statement about the appropriate storage of breast milk is correct?	31 (22.0)	101 (71.6)
Which statement about handling of thawed expressed breast milk is accurate?	22 (15.6)	109 (77.3)

Table 3 compares mean scores of knowledge, attitudes, and practices across variables like gender, occupation, department, professional experience, breastfeeding policy, and WHO training. Males scored higher in knowledge (8.0 ± 1.3) than females (7.1 ± 1.8, p=0.051), while specialists/consultants scored better than nurses (p=0.033). No significant differences in attitude or practice were noted among groups. Departmental affiliation did not significantly impact scores, and interrupted professional experience showed slightly higher knowledge and practice, but not significantly. A breastfeeding policy appeared linked to higher scores, though not statistically significant. Notably, WHO 20-hour training significantly improved practice scores (p=0.024).

**Table 3:** Association of demographic and professional characteristics with knowledge, attitude, and practice scores

*Variables*	*Total n*	*Knowledge mean (SD)*	*Attitude mean (SD)*	*Practice mean (SD)*
**Gender**				
Female	123	7.1 (1.8)	2.9 (1.0)	3.5 (1.7)
Male	18	8.0 (1.3)	3.1 (0.4)	3.1 (1.3)
p		0.051	0.64	0.4
**Occupation**				
Head/charge/midwife/RN	120	7.1 (1.8)	2.9 (1.0)	3.5 (1.7)
Specialist/consultant/CNS	21	8.0 (1.4)	3.2 (0.5)	3.4 (1.4)
p		0.033	0.22	0.9
**Department**				
LR/OBS/GYN/ED	68	7.2 (1.7)	2.9 (0.8)	3.4 (1.7)
Pediatrics-ED/IPW/PICU/NICU	73	7.2 (1.9)	3.0 (1.1)	3.5 (1.7)
p		0.99	0.7	0.84
**Professional experience**				
Interrupted	6	8.2 (2.8)	3.5 (1.0)	4.0 (2.5)
Uninterrupted	134	7.2 (1.7)	2.9 (0.9)	3.4 (1.7)
p		0.26	0.19	0.69
**Breast feeding policy**				
No	4	6.0 (2.4)	2.8 (1.0)	2.5 (0.6)
Yes	137	7.3 (1.8)	3.0 (1.0)	3.5 (1.7)
p		0.16	0.66	0.26
**WHO/UNICEF 20-h BFHI Course**				
No	95	7.2 (1.5)	2.9 (1.0)	3.2 (1.5)
Yes	46	7.3 (2.2)	3.0 (1.0)	3.9 (2.0)
p		0.67	0.46	0.024

Independent t-test, p<0.05 considered for statistical significance.

**Figure 1:**
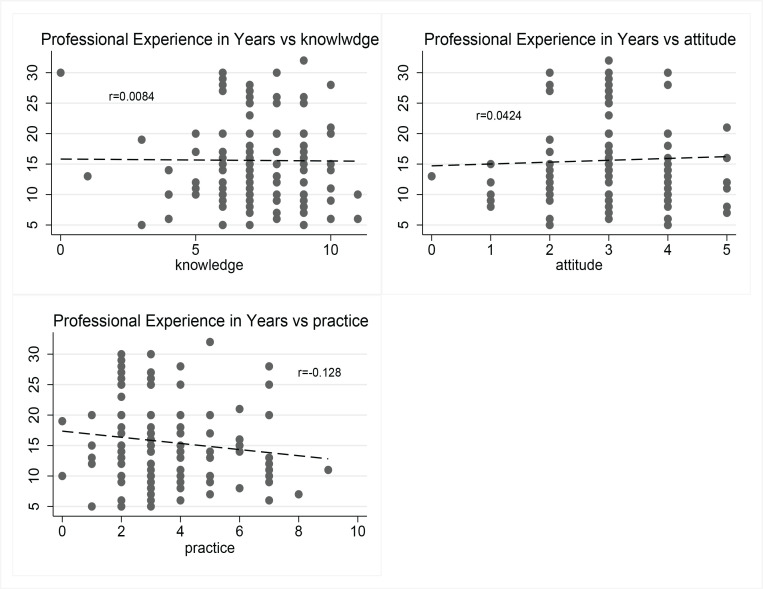
Pearson correlation between professional experience in years and knowledge, attitude, and practice scores

Figure 1 shows the relationship between professional experience and knowledge, attitudes, and practices (KAP) variables. The correlation coefficients indicate weak relationships: knowledge (r = -0.0084, p=0.922) and attitudes (r=0.0424, p=0.626) show negligible positive associations with experience, while practices (r= -0.128, p=0.142) exhibit a weak negative correlation. The trend lines suggest that professional experience does not strongly influence knowledge or attitudes, and there is a slight decline in practices scores with increasing experience.

**Figure 2:**
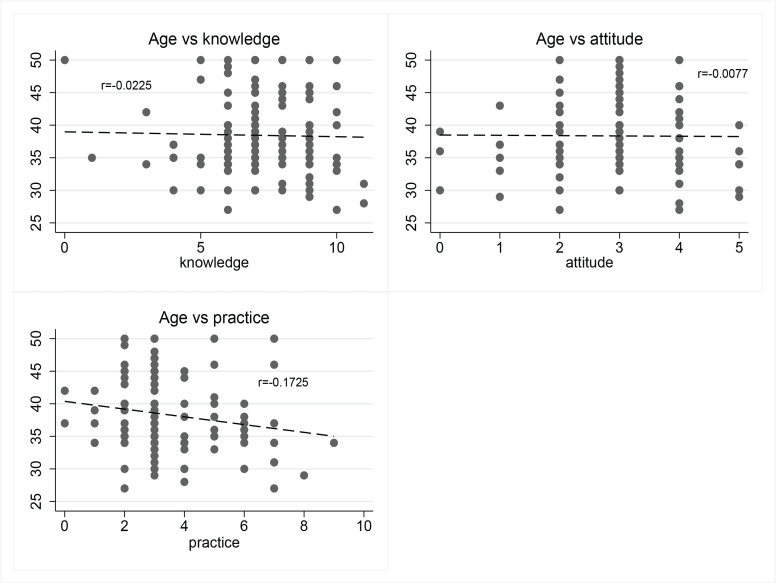
Pearson correlation between age in years and knowledge, attitude, and practice scores

Figure 2 illustrates the relationship between age and knowledge, attitudes, and practices (KAP) variables. The correlation coefficients indicate weak associations: knowledge (r= -0.0225, p=0.792) and attitudes (r= -0.0077, p=0.929) exhibit negligible negative correlations with age, while practices (r= -0.1725, p=0.047) show a slightly stronger but still weak negative correlation. The trend lines suggest that age does not significantly influence knowledge or attitudes, and there is a mild decline in practices scores as age increases.

## DISCUSSION

Breastfeeding is considered an instinct act, but it is also a learned skill that can be easily acquired and practiced^10^. Literature is rich in evidence that supports the tremendous benefits of breastfeeding, especially exclusive breastfeeding (EBF) and the positive impact of the Baby-Friendly Health Initiative (BFHI) on increasing breastfeeding rates, improving and achieving breastfeeding outcomes^12^.

Several studies in literature show that when the BFHI is implemented in the hospital or institution, breastfeeding rates increase and hence, achieving the overall societal benefits from encouraging breastfeeding practice could be obtained^13-17^.

The first few critical hours in the lives of the newborns determine whether they could get EBF or even breastfeeding or not, depending on the level of maternal knowledge and cultural behavior, and the type of support and education they could get to succeed in the process. The breastfeeding process could be hindered or delayed due to many reasons, among which the separation of the mother and the newborn, providing the newborn with pre-lacteal feeds, and giving them needless infant formulas. These actions lead to negative impact on the initiation of breastfeeding and represent major challenges that might bring about interruption to the process^16^. The most critical reason could be attributed to the lack of adequate knowledge of exclusive breastfeeding in some healthcare professionals which could affect the start of the breastfeeding process^16^.

In the current study, the knowledge, attitude, and practical skills of nursing staff on breastfeeding was assessed, a step prior to implementing the Baby-Friendly Hospital Initiative in the hospital.

The majority of the participants in the current questionnaire were female nurses (approximately 90%), which is acceptable as female nurses prefer to work in gynecology, pediatrics and nursing departments than male nurses^18^.

At the beginning of the questionnaire, the participants were asked whether their hospital adopted a breastfeeding policy or not, and the majority agreed that the hospital did (97%). Furthermore, they participants were asked if they have previously received the World Health Organization 20-hours ‘Breastfeeding Promotion and Support in a Baby-Friendly Hospital Course’ or not, and the majority mentioned that they never did (approximately 70%).

Generally, nursing staff in the current work showed good total knowledge scores percentages (68%) about the BFHI. Like our results, Mothukuri et al.^19^ assessed knowledge, attitudes and practices of the nursing staff about implementing the BFHI at their hospital. They reported that most of the nursing staff had good knowledge about EBF and about the basics of the 10 steps of the BFHI^19^. Similar results were reported in India in 2019, where around 80% of nurses showed moderately adequate knowledge of the BFHI^20^. Our positive knowledge results may be attributed to the managerial role in providing training and knowledge for staff regarding BFHI.

Regarding the knowledge, the questionnaire comprised 10 questions to assess staff’s knowledge. When they were asked about the correct definition of breastfeeding, 60% of participants gave correct answers. On asking about the global recommendation for duration of exclusive breastfeeding, 70% of the staff gave the correct answer on exclusively fed for the first 6 months of their early lives. In a similar study, nursing staff’s knowledge on breastfeeding, around 70% of their nursing staff had good knowledge on EBF, and gave the correct definition of breastfeeding. Similar positive responses were recorded in the study when questioning about the importance of immediate and sustained mother-baby skin-to-skin contact after birth. These results were in accordance with previous work.

The staff answered correct responses in high percentages as 72%, 85% and 95% on the questions asking about the meaning of responsive or demand feeding, suitable time to interrupt skin-to-skin contact within the first 2 hours after birth, and reasons to support 24-hour rooming-in breastfeeding, respectively. Similar results to questionnaire scores were reported by Daniels and Jackson^21^ in their similar BFHI assessment study in Cape Town, where they reported that around 60% of their staff defined rooming-in correctly, and generally more than 50% were able to define baby-friendly care practices.

Unexpectedly, the majority of our nursing staff failed to give the correct answer regarding the global recommendation for how long a baby should be breastfed (75%). In addition, they could not identify newborn’s behaviors that express instinctually before latching to mother’s breast (62%).

The participants gave different answers to reasons why skin-to-skin contact should not be uninterrupted, but the majority failed to identify the correct reason. Around 40% answered that the newborn’s blood glucose would increase, and the 10% mentioned decrease in the cortisol’s level and 3% thought that the baby’s tone would decrease. Only 46% stated about infant hypothermia.

A recent study in Singapore in 2022, assessed nurses’ knowledge attitude and practice on breastfeeding after receiving the 20-hours BFHI training program in a tertiary hospital. They reported that participants showed greater breastfeeding knowledge, and improved awareness about the initiative goals post education^16^. Another study suggested that arranging and calling for programs like BFHI to encourage breastfeeding practices necessitates updating and enhancing knowledge of the healthcare team about these practices.

Regarding the attitude section of the current questionnaire, 60% of participants showed positive attitudes to the BFHI represented in correct answers to the questions. In accordance with our results, previous studies have also positive attitudes towards the nursing staff along with adequate knowledge towards breastfeeding^20^.

The answers of our respondents in the attitude section were correct pertaining to: how should a direct care provider respond if he/she was offered rewards by an infant-feeding company representative (79.4%), reasons why a healthcare provider should refuse any financial or material inducements (80.9%), and how healthcare staff could influence facilities to avoid promotion of feeding bottles or teats (83.7%). But their attitude results were negative in 2 of the questions, the first was asking about how to respond if offered informational materials provided by an infant feeding company representative (13.5%) and the second negative attitude that they could not provide best answers to a worried new mother about using bottle feeds to provide good sleep to her infant (27.7%).

In the practice section of the present work, the percentage of staff practicing the BFHI was relatively low (45%). Similarly, Mothukuri et al.^19^ reported that they still had a lack of total awareness of some essential practices of the BFHI in the results of their questionnaire though having high knowledge scores.

In the present work, the participants showed correct practice responses in some major practice cornerstones, as they knew sequence when placing the infant skin-to-skin with the mother (61.7%) and the key points for effective positioning baby at breast (72.3%).

Regarding the appropriate storage of breast milk according to the WHO recommendations, most of our participants did not give the correct answer (71.6%). This highlights the importance of conducting comprehensive and regular training for the healthcare providers pertaining to breastfeeding and the BFHI concepts.

On correlating the knowledge, attitude, and practice scores, we found significant positive correlation between knowledge and practice scores (r=0.35, p<0.01). Another positive correlation was found between the attitude score and the practice score (r=0.402, p<0.01).

This is consistent with previous studies that showed significantly improved BFHI practicing with high level of basic knowledge of breastfeeding^10,20^. Another study reported that although they showed initial positive knowledge results, their staff still had a lack of knowledge and practical skills.

The change that is required for implementing BFHI is hard due to many reasons. As stated in literature mothers’ education was a cornerstone to be able to apply the goals of BFHI. No mother’s education is possible unless we educate the healthcare providers.

Interventions to educate healthcare providers have been proven to be significantly successful in boosting mothers to practice EBF. However, these interventions might not be successful in some developing countries^22^. In our study, training the healthcare providers has proven to be efficient in raising their awareness and knowledge about the BFHI and could enhance helping mothers achieve the EBF goals with their children.

Previous studies showed a notable improvement in breastfeeding knowledge, attitudes, and practices, as well as the implementation of BFHI policies following interventions focused on educating healthcare staff.

The health authority in the State of Qatar encourages hospitals to implement the BFHI policy since 2016, but not all hospitals gained the accreditation^9^. Chehab et al.^9^ described several obstacles for achieving implementation in Qatar, the most significant was the relatively short maternity leaves.

In 2017, the WHO released a guideline tagged ‘*Protecting, promoting and supporting breastfeeding in facilities providing maternity and newborn services*’ which was updated in 2018. This guideline encouraged governmental entities to support breastfeeding and BFHI practice and to integrate the program in its healthcare system^2^.

The WHO and the UNCEF in their 2018 implementation guidance recommended several steps to implement the BFHI and to enable national application and ensure endurance of the BFHI practice over time. The guidance stressed on ‘integrating the protection, promotion and support of breastfeeding more fully into the healthcare system, including in private and public facilities. The modifications and increased feasibility serve the purpose of increasing newborns’ access to breastfeeding in all facilities, not only a select few’^2^.

As stated earlier, while breastfeeding is the most natural means for nourishing and nurturing an infant, it does not come naturally to all mothers and infants. It requires a combination of appropriate early care practices and ongoing support by skilled health professionals.

The Baby-Friendly Hospital initiative aims to ensure that mothers and newborns receive ‘timely and appropriate care before and during their stay in a facility providing maternity and newborn services …’^2^.

A core purpose of the BFHI is to guarantee the competency of health professionals and managers in the implementation of the Ten Steps. The 2018 revision of the Ten Steps underscores that all direct care providers should have the competencies needed to ensure care is delivered consistently and ethically.

Sufficient knowledge, skills and attitudes to support breastfeeding are essential for the provision of safe, evidence-based, compassionate care. Staff training and/or formal education are still important in gaining knowledge and technical skills. Moreover, all direct care providers working in facilities that provide maternity and newborn services are expected to demonstrate their competencies in relevant aspects of breastfeeding counselling and support.

The implementation of the Ten Steps in maternity facilities requires knowledge, skills and attitudes beyond just those needed for basic breastfeeding support. BFHI involves ethical aspects of care and services to all mothers, supported by a facility’s policy and respect of the International Code of Marketing of Breast-milk Substitutes. Direct care providers must support women’s informed decisions related to their infant’s nutrition and well-being, which encompasses more than clinical breastfeeding support.

BFHI is about actively participating in providing enabling environments for sustainable implementation within the facility so that all mothers and infants receive the evidence-based, individualized and compassionate care they deserve from all direct care providers working towards the same goal.

In the present work, the hospital offers a 20-hours course on the BFHI practice to all healthcare providers, and it is repeated 3 to 4 times around the year, yet a limited number of staff attend the course and this yields small group of educated team members.

These educated staff about the concepts of breastfeeding and BFHI, despite being few, they had a great impact on raising the overall knowledge and awareness of our staff about BFHI concepts. This reflects the significant effect of educating healthcare staff on obtaining goals of the BFHI.

Similarly in their study, Fok et al.^16^ assured that implementing the 20-hour BFHI educational program on their nursing staff significantly affected knowledge, attitude, and assurance of the staff in breastfeeding and BFHI practices. In addition, they advised that the organizational policies and manpower needs should be covered to prevent these factors from representing an obstacle on offering breastfeeding consultations to mothers^16^.

Our study highlights the importance of breastfeeding in general and shows the relatively high awareness of the healthcare staff at our hospital in Qatar regarding the importance of breastfeeding and EBF on mothers, children, and the whole country, pertaining general health of citizens and the future economic impact of the initiative. In addition, our current work showed the readiness of the staff to implement the BFHI and gain accreditation to our hospital, after then inspire other hospitals to apply for the BFHI.

Finally, it is crucial to note the significant cultural differences that may influence training programs, especially in multicultural regions like the Middle East. By understanding specific cultural norms and religious beliefs regarding breastfeeding, educational programs can be tailored to be more inclusive, helping to increase effectiveness.

In conclusion, this study’s findings reveal the critical need for targeted educational interventions that not only enhance knowledge and skills but also connect with the diverse backgrounds and experiences of healthcare professionals to improve breastfeeding practices and outcomes.

## CONCLUSIONS

Our study indicates the critical need for comprehensive breastfeeding training programs that extend beyond theoretical knowledge to emphasize practical skills among healthcare providers. Despite the understanding of breastfeeding principles, significant gaps in practical application remain, highlighting the importance of targeted educational interventions. The positive impact of the WHO 20-hour training on practical application scores indicates a pathway for improvement. Additionally, while demographic factors show some influence on knowledge scores, the overall attitudes and practices require further enhancement. Future research should explore the effectiveness of training in diverse cultural contexts, particularly in the Middle East, to strengthen breastfeeding support in clinical settings.

## Supplementary Material



## Data Availability

The data supporting this research are available from the authors on reasonable request.
