# Corrigendum: Healthcare providers insights on the Baby-Friendly Hospital Initiative: A cross-sectional study in Qatar

**DOI:** 10.18332/ejm/211648

**Published:** 2025-10-23

**Authors:** Jussara D. S. Brito, Kalpana Singh, Laura Falcon, Soad Elkhaligy, Tamara Alshdafat, Salwa Alrawaili, Lolwa Alansari

**Affiliations:** 1 Al Wakra Hospital, Hamad Medical Corporation, Al Wakra, Qatar; 2 Nursing Researcher Department, Hamad Medical Corporation, Doha, Qatar; 3 Women’s Wellness & Research Centre, Hamad Medical Corporation, Doha, Qatar; 4 Gardenia Medical Center, Doha, Qatar

**Keywords:** Baby-Friendly Hospital Initiative, KAP analysis, breastfeeding, healthcare

Corrigendum on:

Healthcare providers insights on the Baby-Friendly Hospital Initiative: A cross-sectional study in Qatar

By Jussara D. S. Brito, Kalpana Singh, Laura Falcon, Soad Elkhaligy, Tamara Alshdafat, Salwa Alrawaili, Lolwa Alansari

European Journal of Midwifery, Volume 9, Issue June, Pages 1-9.

Publish date: 12 June 2025

DOI: https://doi.org/10.18332/ejm/203687

In the originally published version of the article, the information below was missing from the Funding section:

This research was supported by the Medical Research Center of Hamad Medical Corporation. This is corrected also online.

